# Tcf1 Sustains the Expression of Multiple Regulators in Promoting Early Natural Killer Cell Development

**DOI:** 10.3389/fimmu.2021.791220

**Published:** 2021-11-30

**Authors:** Juanjuan Liu, Zhao Wang, Shanshan Hao, Fang Wang, Yingpeng Yao, Yajiao Zhang, Yanyi Zhao, Wenhui Guo, Guotao Yu, Xiaohan Ma, Jingjing Liu, Feng Chen, Shunzong Yuan, Youmin Kang, Shuyang Yu

**Affiliations:** ^1^ State Key Laboratory of Agrobiotechnology, College of Biological Sciences, China Agricultural University, Beijing, China; ^2^ Central Laboratory, School of Stomatology, Peking University, Beijing, China; ^3^ Department of Hematology, the Fifth Medical Center of People’s Liberation Army (PLA) General Hospital, Beijing, China

**Keywords:** Tcf1, NK cell, NKP, mice, development, targets

## Abstract

T cell factor 1 (Tcf1) is known as a critical mediator for natural killer (NK) cell development and terminal maturation. However, its essential targets and precise mechanisms involved in early NK progenitors (NKP) are not well clarified. To investigate the role of Tcf1 in NK cells at distinct developmental phases, we employed three kinds of genetic mouse models, namely, *Tcf7* ^fl/fl^ *Vav* ^Cre/+^, *Tcf7* ^fl/fl^ *CD122* ^Cre/+^ and *Tcf7* ^fl/fl^ *Ncr1* ^Cre/+^ mice, respectively. Similar to Tcf1 germline knockout mice, we found notably diminished cell number and defective development in BM NK cells from all strains. In contrast, *Tcf7* ^fl/fl^ *Ncr1* ^Cre/+^ mice exhibited modest defects in splenic NK cells compared with those in the other two strains. By analyzing the published ATAC-seq and ChIP-seq data, we found that Tcf1 directly targeted 110 NK cell-related genes which displayed differential accessibility in the absence of Tcf1. Along with this clue, we further confirmed that a series of essential regulators were expressed aberrantly in distinct BM NK subsets with conditional ablating Tcf1 at NKP stage. *Eomes*, *Ets1*, *Gata3*, *Ikzf1*, *Ikzf2*, *Nfil3*, *Runx3*, *Sh2d1a*, *Slamf6*, *Tbx21*, *Tox*, and *Zeb2* were downregulated, whereas *Spi1* and *Gzmb* were upregulated in distinct NK subsets due to Tcf1 deficiency. The dysregulation of these genes jointly caused severe defects in NK cells lacking Tcf1. Thus, our study identified essential targets of Tcf1 in NK cells, providing new insights into Tcf1-dependent regulatory programs in step-wise governing NK cell development.

## Highlights

- Excluding the effects from microenvironment *via* conditional ablation of Tcf1 in NK cells at distinct developmental stages.
- Elucidating stage-specific roles of Tcf1 in the step-wise development of NK cells.
- Deciphering the regulatory network of Tcf1 in early NK progenitors.
- Bridging Tcf1-dependent NK regulators and the defective development of NK cells lacking Tcf1.

## Introduction

Natural killer (NK) cells are one of the founding members of the innate lymphoid cell (ILC) family (1), which play essential roles in primary immune response *via* their cytotoxicity or cytokine production activities without prior sensitization (2). NK cells originate from lymphoid-primed multipotent progenitors (LMPPs) and common lymphoid progenitors (CLPs) (3), which are lymphoid restricted multipotent cells that also generate other lymphocytes, such as B, T, dendritic cells (DCs), and ILCs (4). The upregulation of CD122, β-chain of the IL-15 receptor, is associated with the commitment to the NK cell lineage (5, 6), thereof the NK cell progenitors (NKPs) can be generally defined by expressing CD122 with the activating receptor NKG2D within lineage negative population of bone marrow (BM) (7). This NKP population has been further identified by the co-expression of 2B4 (CD244), CD27 (Tnfrsf7), and surface lymphocyte activation molecule (SLAM)-family receptors with the majority of these cells also expressing IL-7Ra (8). Once turning on expression of the activating NK cell receptors CD49b and NK1.1/NCR1, NKPs step in a sequential process of maturation and gain NK cell functions. Accompanying with the dynamic expression of CD27 and CD11b, the NK1.1^+^ or NCR1^+^ murine NK cells can be further subdivided into three developmental phases, namely, immature NK (iNK, CD27^+^CD11b^−^) stage, transitional double positive (DP, CD27^+^CD11b^+^) stage, and terminal mature NK (mNK, CD27^−^CD11b^+^) stage (9), which also express high level of KLRG1 (10).

The developmental process of NK cells is tightly controlled by stage-specific transcription factors, including those of which are essential for the signaling downstream of IL-15 receptors. The signal transducer and activator of transcription proteins STAT5a and STAT5b are critical in NK cell development *via* sustaining survival, proliferation, and maturation (11, 12). Ets-1 and PU.1 regulate the transition from CLPs to NKPs (13, 14). Nfil3 is induced in CLPs and its expression is crucial in the induction of CD122 and differentiation towards the ILC/NK cell lineages *via* directing Eomes and Id2 expression (15). Over the years, accumulating pieces of evidence have showed the essential roles of Id2, Eomes, Ets1, Gata3, Ikzf1, Ikzf2, Nfil3, RunX3, Sh2d1a, Slamf6, Spi1, Tbx21, Tox, and Zeb2 in NK cell development and maturation at distinct stages, respectively (13, 14, 16–24). Despite performing a series of past studies which focused on the transcriptional regulation on NK cells by using different mouse models (25), the precise mechanisms of NK cell development are not entirely understood (26).

Given NK cells are regarded as the innate counterpart to CD8^+^ T cells, a series of transcription factors governing early developmental programs and activation of NK cells parallel to those of CD8^+^ T cells, but the refined mechanisms are quite distinct (27). As a smart T cell regulator, Tcf1 (encoded by *Tcf7*) also functions as a key role in modulating early NK cell development and function (28–32). Tcf1-deficient NK cells dysregulate the expression of multiple receptors which impair their properly license and produce high level of GZMB which is harmful to their viability (30, 31). Despite the negligible expression level of Tcf1 in cytotoxic CD27^-^CD11b^+^ mNK, Tcf1 deficiency skews NK subpopulation toward terminal maturation (31, 32). *Tcf7* ^−^ *^/^* ^−^ mice show reduction of their NK cell numbers and aberrant development in both BM and peripheral tissues, whereas conditional ablation of Tcf1 with *Ncr1*-Cre beyond NKP stage exhibits modest phenotypic defects in both NK cell numbers and development (31, 32). Although previous studies implied that Tcf1 functions as an indispensable regulator in early stage development of NK cells, the precise regulatory mechanisms and essential targets downstream of Tcf1 are still so far poorly understood.

In this study, we developed *Tcf7* ^fl/fl^ *Vav* ^Cre/+^, *Tcf7* ^fl/fl^ *CD122* ^Cre/+^, and *Tcf7* ^fl/fl^ *Ncr1* ^Cre/+^ mice to investigate the stage specific roles of Tcf1 in NK cell development. Combined with published high-throughput data, we focused on the targets downstream of Tcf1 in distinct developmental phases. Our data demonstrate that Tcf1 serves as a smart stage-specific regulator of transition from NKPs to functional maturation and provide critical insights into the potential Tcf1 dependent regulatory programs in early NK cell development.

## Materials and Methods

### Mice

All mice used in this study were between 7 and 10 weeks of age on a fully C57BL/6J background. *Vav*-Cre and *Tcf7* ^fl/fl^ mice (from Jackson Laboratories) were maintained as previously described (33, 34). *CD122*-Cre mice were kindly provided by Dr. Zhongjun Dong (Tsinghua University, China). *Ncr1*-Cre mice were purchased from the Biocytogen Pharmaceuticals company (Beijing, China). Mice were housed in specific pathogen-free conditions under controlled temperature (22 ± 1°C) and exposed to a constant 12-hour light/dark cycle. All institutional and national guidelines for the care and use of laboratory animals were followed and all animal protocols used in this study were approved by the Institutional Animal Care and Use Committee at China Agricultural University.

### Cell Isolation, Staining and Flow Cytometry

Single-cell suspensions were isolated and prepared from bone marrow (BM), spleen (SP), or peripheral blood cells (PBCs) as previously described (35). For surface staining, cells were stained with fluorescence-conjugated antibodies in FACS buffer (PBS + 2% FBS). For intranuclear staining of transcription factors Tcf1, cells were fixed and permeabilized with Cytofix/Cytoperm Fixation/Permeabilization Solution Kit (554714, BD Biosciences) following the manufacturer’s instruction as previously described (36). The following fluorochrome-labeled monoclonal antibodies from eBioscience were used: CD3e (145-2C11), CD122 (TM-b1), CD49b (DX5), NK1.1 (PK136), CD11b (M1/70), CD27 (LG.7F9), Ly49A (A1), Ly49D (eBio4E5), Ly49E/F (CM4), Ly49G2 (eBio4D11), and Ly49H (3D10). Samples were acquired with FACSVerse or FACS LSRFortessa II (BD Biosciences) following the manufacturer’s instruction. The flow cytometry data were analyzed with FlowJo software (Version 10.4.0, Tree Star, Inc.). For cell sorting, cells were stained with corresponding fluorescence-conjugated antibodies and subjected to sorting on a FACS Aria II (BD Biosciences) as previously described (37).

### RNA Extraction, Reverse Transcription and Quantitative Real-Time PCR

Total RNA was extracted from sorted cells using the RNeasy Mini Kit (Qiagen) or RNeasy Micro Kit (Qiagen) followed by cDNA synthesis with FastQuant RT Kit (Tiangen) as previously described (36). Quantitative RT-PCR was carried out with SuperReal PreMix Plus SYBR Green (Tiangen) on a CFX96 Connect™ Real-Time System (Bio-Rad). The results were normalized to the expression of housekeeping gene *Gapdh* transcript. Differences in expression levels were calculated according to the 2^−ΔCT^ method. All primers used are listed in **Supplementary Table 1**.

### Apoptosis Analysis

Single-cell suspensions were stained with indicated surface antibodies, and then resuspended in Annexin V binding buffer (BD Biosciences). Apoptosis assays were performed by staining cells with Annexin V and 7-aminoactinomycin D (7-AAD) (BD Biosciences) following the manufacturer’s instruction. The stained cells were acquired on a FACSVerse or FACS LSRFortessa II equipment (BD Biosciences).

### RNA-Seq Data Analysis

For NK related genes transcriptional profiles, RNA-seq of BM and splenic NK cells subpopulations were originated from the Gene Expression Omnibus with accession no. GSE109125. The normalized counts were generated with the DESeq2 package in R (4.1.4). The clustered heatmap was performed using the “pheatmap” package in R (4.1.4) with the scale = “row” parameter.

### ChIP-Seq Data Analysis

ChIP-seq of splenic CD8^+^ T cells was originated from the Gene Expression Omnibus with accession no. GSE73239. Quality assessment of raw reads was assessed by FastQC (0.11.9) and adaptors were removed using Trim_galore (0.6.3). The processed data were mapped to the mouse reference genomes (mm10) which were downloaded from the UCSC repository using Bowtie (2.1.0). The aligned results converted to bigWig format by Bedtools (2.26.0) were visualized on the Integrated Genome Viewer from the Broad Institute. MACS (2.1.1) was used to call binding sites (peaks) relative to Input libraries with the p-value threshold set as 1 × 10^−5^. Finally, called peaks were annotated against the mouse reference genomes (mm10) in HOMER.

### ATAC-Seq Data Analysis

Given that there are no published Tcf1-dependent ATAC-seq in NK cells, we have initially compared ATAC-seq dataset (GSE99159) in CD4^+^CD8^+^ double positive (DP) thymocytes and several datasets (GSE119839, GSE144382, and GSE139024) in CD8^+^ cells which are associated with the Tcf1 regulation. We eventually chose the ATAC-seq data (GSE99159) of DP thymocytes, which were a high-quality data to reflect the altered chromosome accessibility in the absence of Tcf1 during naïve lymphocyte development. The quality assessment of raw reads was assessed by FastQC (0.11.9) and adaptors were removed using Trim_galore (0.6.3). The processed data were mapped to the mouse reference genomes (mm10) which were downloaded from the UCSC repository using Bowtie (2.1.0). Subsequently, the PCR duplication was eliminated by Sambamba (0.8.1). The aligned results converted to bigWig format by Bedtools (2.26.0) were visualized on the Integrated Genome Viewer from the Broad Institute. Peak calling was performed using MACS (2.1.1) with default parameter and regions of differential enrichment being tested with the DiffBind package in R (4.1.4).

### Venn Analysis

We generated the NK cell-related gene set ourselves for overlapping analysis with Tcf1 regulated genes. In brief, the NK cell-related genes were retrieved genes in part from the existing gene sets associated with NK cells on the Gene Ontology Resource website (http://geneontology.org/), GSEA-MSigDB (http://www.gsea-msigdb.org/), and a linked data server (http://www.ontobee.org/), and combined a number of known-genes involved in NK cell development and/or function based on published literatures. The NK cell-related genes, Tcf1 binding gene set, and differentially accessible genes set were used for Venn analysis. Venn diagram analysis was performed using online software at the following URL: http://bioinformatics.psb.ugent.be/webtools/Venn/.

### Statistical Analysis

Statistical analysis was performed using Prism 7.0 (GraphPad Software) and the error bar was shown as means ± SD. The data were shown as mean ± SD in all graphs, and statistical differences were calculated using a one-tailed unpaired Student’s t-test, unless otherwise specified. The statistically significant measurements are marked as follows: *P <0.05, **P <0.01, and ***P <0.001.

## Results

### Generation of Distinct Mouse Models With Conditional Ablation of Tcf1 in NK Cells at Three Developmental Phases

**Figure 1:**
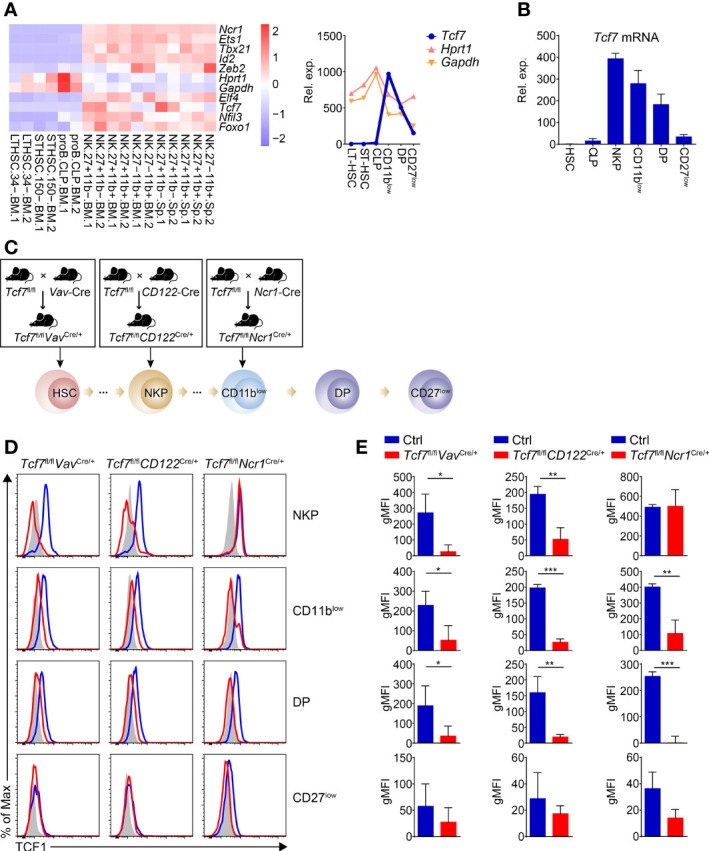
Analyzing dynamic expression of *Tcf7* in NK subsets and detecting Tcf1 deletion efficiency in NK cells from the three conditional targeting mouse models. **(A)** Left: Heatmap showing the mRNA abundance of *Tcf7* compared with several NK signature genes in various cell subsets by published RNA-seq data (GSE109125); Right: A line chart showing normalized counts of *Tcf7* expression trend in continuous developmental stages of NK cells in comparison with housekeeping genes *Gapdh* and *Hprt1*. **(B)** Analysis of the relative *Tcf7* abundance by qPCR in HSC, CLP, NKP, CD11b^low^ (CD3e^−^NK1.1^+^CD11b^−^CD27^+^), DP (CD3e^−^NK1.1^+^CD11b^+^CD27^+^), and CD27^low^ (CD3e^−^NK1.1^+^CD11b^+^ CD27^-^) cells. *Gapdh* was arbitrarily set to 1,000 and the relative abundance of *Tcf7* in each subset was normalized, accordingly (n ≥3 for each group). **(C)** Schematic graphs showing the strategies for generating mouse models. *Tcf7* ^fl/fl^ mice were mated with *Vav*-Cre, *CD122*-Cre or *Ncr1*-Cre transgenic mice, respectively. The Cre recombinase initially expressed in each developmental stage was indicated with a black arrow. **(D)** The expression of Tcf1 in the indicated NK subsets was analyzed with flow cytometry. Representative histogram plots showed the expression of Tcf1 with shadow (Isotype control), blue line (littermate controls), and red line (conditional knock out mice as indicated), respectively. **(E)** Cumulative data of geometric mean fluorescence intensity (gMFI) from panel **(D)** are shown, accordingly (n = 3 for each group). The gMFIs were calculated by deducting the value of Isotype controls from those of either Ctrl or knockout mice. Statistical significance was determined by one-tailed Student’s t-test. *P < 0.05, **P < 0.01, and ***P < 0.001. Data are means ± SD.

To gain insights of the importance of Tcf1 (encoded by *Tcf7*) in the entire process of NK cell development, we went over the expression of *Tcf7* from hematopoietic stem cells (HSC) to distinct NK cell subsets along with developmental phases by analyzing published data (GSE109125) from the ImmGen database. Compared with other classic NK regulators, *Tcf7* exhibits dynamic expression and upregulates its expression beyond common lymphoid progenitor (CLP) stage (**Figure 1A**). Meanwhile, we found it reached a peak in CD11b^low^ (CD3e^−^NK1.1^+^CD27^+^CD11b^−^) iNK compartment in both bone marrow (BM) and spleen (SP) at a relatively high level in comparison with housekeeping gene expression of *Gapdh* or *Hprt1* (**Figure 1A**). The expression of *Tcf7* in distinct NK subpopulations was further confirmed by qPCR, and its expression trend was in line with high-throughput data, exhibiting high abundances in NKP (CD3e^−^CD122^+^ DX5^−^NK1.1^−^) and CD11b^low^ stages (**Figure 1B**). These data collectively implied the potential roles of Tcf1 in dynamically regulating early NK cell development.

Recently, Jeevan-Raj et al. have identified that Tcf1 intrinsically regulates the development of NK cells and guarantees their survival, but the precise mechanism is still unclear (31). To further elucidate stage-specific roles of Tcf1 in NK cell development, we employed three genetic models by crossing *Tcf7* ^fl/fl^ mice with *Vav*-Cre (38), *CD122*-Cre (39) or *Ncr1*-Cre (40) transgenic mice to conditionally inactivate Tcf1 at HSC, NKP or iNK stage, respectively (**Figure 1C**). The deletion efficiency was further confirmed by intracellular staining (ICS) in relative subsets of NK cells (**Figures 1D, E**). Tcf1 was efficiently deleted at protein level in NKP, CD11b^low^, and DP (CD3e^−^NK1.1^+^CD27^+^CD11b^+^) stages from both *Tcf7* ^fl/fl^ *Vav* ^Cre/+^ and *Tcf7* ^fl/fl^ *CD122* ^Cre/+^ mice (**Figures 1D, E**). The comparable efficiency of Tcf1 deletion was also detected in CD11b^low^, and DP subpopulations excluding NKP cells from *Tcf7* ^fl/fl^ *Ncr1* ^Cre/+^ mice since the Cre recombinase is only expressed in Ncr1^+^ cells (**Figures 1D, E**). The protein level of Tcf1 in terminal mNK (CD27^low^, CD3e^−^NK1.1^+^CD27^−^CD11b^+^) cells was not significantly reduced in all three conditional knockout strains owing to its negligible expression at this stage (**Figures 1D, E**).

### Tcf1 Deficiency at HSC Stage Severely Impairs NK Cell Development

**Figure 2:**
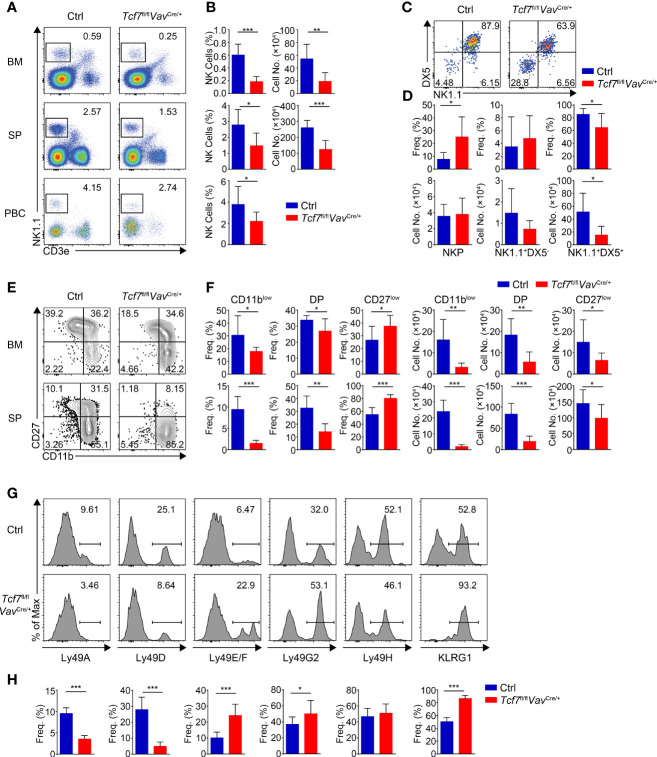
Ablating Tcf1 at HSC stage impairs NK cell development. **(A)** Flow cytometry analysis of BM, SP, and PBCs from Ctrl and *Tcf7* ^fl/fl^ *Vav* ^Cre/+^ mice. Representative pseudocolor plots showed total NK (CD3e^-^NK1.1^+^) cells from indicated tissue. **(B)** The frequency (left) and absolute numbers (right) of the NK cells from panel **(A)** are shown, accordingly (n ≥5 for each group). **(C)** Flow cytometry analysis of NKP cells in BM from Ctrl and *Tcf7* ^fl/fl^ *Vav* ^Cre/+^ mice. The CD3e^−^CD122^+^ cells were further analyzed by using NK1.1 and DX5 staining. Representative pseudocolor plots showed NKP (DX5^-^NK1.1^−^), DX5^−^NK1.1^+^, and DX5^+^NK1.1^+^ cells. **(D)** The percentages (upper) and absolute numbers (lower) of NKP, DX5^−^NK1.1^+^, and DX5^+^NK1.1^+^ cells from panel **(C)** are shown, accordingly (n ≥6 for each group). **(E)** Flow cytometry analysis of CD3e^−^NK1.1^+^ cell subsets from Ctrl and *Tcf7* ^fl/fl^ *Vav* ^Cre/+^ mice. The CD3e^−^NK1.1^+^ cells from BM or SP were further analyzed by using CD11b and CD27 staining. Representative contour plots showed CD11b^low^ (CD11b^−^CD27^+^), DP (CD11b^+^CD27^+^) and CD27^low^ (CD11b^+^ CD27^−^) NK cells. **(F)** The frequency (left) and absolute numbers (right) of CD11b^low^, DP and CD27^low^ NK cells from panel **(E)** are shown, accordingly (n ≥6 for each group). **(G, H)** Representative histograms **(G)** showed the expression of Ly49A, Ly49D, Ly49E/F, Ly49G2, Ly49H, and KLRG1 in SP NK cells from Ctrl and *Tcf7* ^fl/fl^ *Vav* ^Cre/+^ mice. The percentage of each receptor is shown in panel **(H)**, accordingly (n ≥6 for each group). All data represent at least three independent experiments. Statistical significance was determined by one-tailed Student’s t-test. *P < 0.05, **P < 0.01, and ***P < 0.001. Data are means ± SD.

We first examined the NK cells in BM, SP, and peripheral blood cells (PBCs) from *Tcf7* ^fl/fl^ *Vav* ^Cre/+^ mice. Compared with their wildtype littermate controls (Ctrls), the frequency and numbers of total NK1.1^+^ cells were substantially reduced in the absence of Tcf1 (**Figures 2A, B**). We next analyzed the early stage development of NK cell in BM with CD122 and DX5 staining. The frequency of NKP subset was notably increased, whereas the percentage of CD3e^−^CD122^+^DX5^+^NK1.1^+^ cells was remarkably decreased in BM from *Tcf7* ^fl/fl^ *Vav* ^Cre/+^ mice (**Figures 2C, D**). Although the absolute numbers of CD3e^−^CD122^+^DX5^+^NK1.1^+^ cells were severely diminished, there was no significant difference in the numbers of NKP and CD3e^−^CD122^+^DX5^−^NK1.1^+^ cells. Further, we found that the frequency and numbers of CD11b^low^ cells and transient DP NK cells were notably impaired (**Figures 2E, F**). Although the frequency of CD27^low^ cells was substantially increased in both BM and SP from *Tcf7* ^fl/fl^ *Vav* ^Cre/+^ mice, the cell numbers were remarkably reduced owing to diminished total numbers of NK1.1^+^ cells (**Figures 2E, F**). Given that Tcf1 plays a key role in the establishment of a repertoire of MHC class I-specific Ly49 receptors (28, 41), we then analyzed the expression of Ly49 receptors in splenic NK cells. The reduced expression of Ly49A and Ly49D and the increased expression of Ly49E/F and Ly49G2 were observed in splenic NK1.1^+^ cell from *Tcf7* ^fl/fl^ *Vav* ^Cre/+^ mice, whereas the expression of Ly49H was not altered in comparison with those from Ctrls (**Figures 2G, H**). In addition, the terminal mature receptor KLRG1 was significantly more abundant (**Figures 2G, H**). These findings collectively indicated that conditional ablation of Tcf1 at the HSC stage resulted in a blockade from NKP to iNK transition and more abundant terminal maturation of NK cell development, which were in accordance with the phenotypes in NK cells from Tcf1 germline knockout mice (31).

### Conditional Ablation of Tcf1 at NKP Stage Exhibits Similar Phenotypic Defects in NK Cell Development of *Tcf7* ^fl/fl^ *Vav* ^Cre/+^ Mice

**Figure 3:**
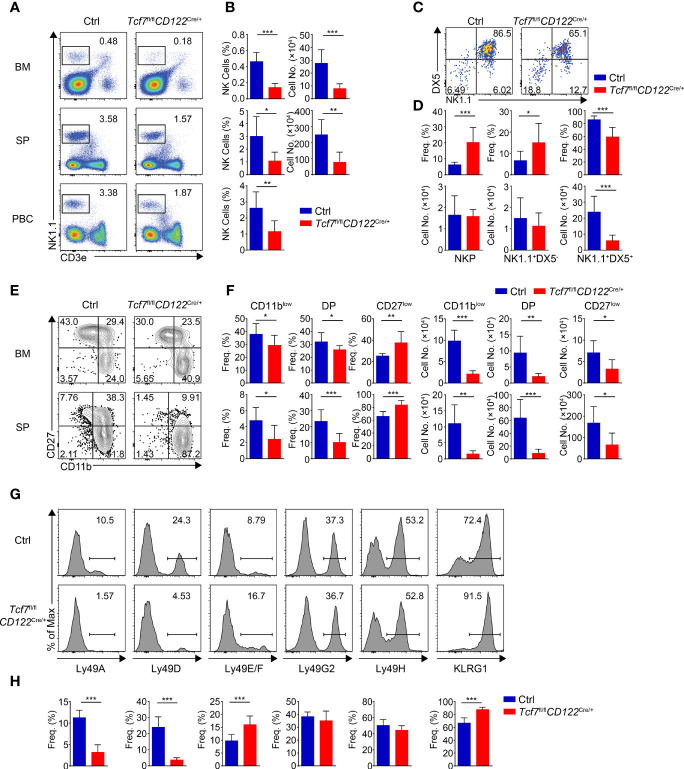
Ablating Tcf1 at NKP stage results in severe defects in NK cell development. **(A)** Flow cytometry analysis of BM, SP, and PBCs from Ctrl and *Tcf7* ^fl/fl^ *CD122* ^Cre/+^ mice. Representative pseudocolor plots showed total NK (CD3e^−^NK1.1^+^) cells from indicated tissue. **(B)** The frequency (left) and absolute numbers (right) of the NK cells from panel **(A)** are shown, accordingly (n ≥5 for each group). **(C)** Flow cytometry analysis of NKP cells in BM from Ctrl and *Tcf7* ^fl/fl^ *CD122* ^Cre/+^ mice. The CD3e^−^CD122^+^ cells were further analyzed by using NK1.1 and DX5 staining. Representative pseudocolor plots showed NKP (DX5^−^NK1.1^−^), DX5^−^NK1.1^+^, and DX5^+^NK1.1^+^ cells. **(D)** The percentages (upper) and absolute numbers (lower) of NKP, DX5^−^NK1.1^+^, and DX5^+^NK1.1^+^ cells from panel **(C)** are shown, accordingly (n ≥6 for each group). **(E)** Flow cytometry analysis of CD3e^−^NK1.1^+^ cell subsets from Ctrl and *Tcf7* ^fl/fl^ *CD122* ^Cre/+^ mice. The CD3e^−^NK1.1^+^ cells from BM or SP were further analyzed by using CD11b and CD27 staining. Representative contour plots showed CD11b^low^ (CD11b^−^CD27^+^), DP (CD11b^+^CD27^+^), and CD27^low^ (CD11b^+^ CD27^−^) NK cells. **(F)** The frequency (left) and absolute numbers (right) of CD11b^low^, DP and CD27^low^ NK cells from panel **(E)** are shown, accordingly (n ≥5 for each group). **(G, H)** Representative histograms **(G)** showed the expression of Ly49A, Ly49D, Ly49E/F, Ly49G2, Ly49H, and KLRG1 in SP NK cells from Ctrl and *Tcf7* ^fl/fl^ *CD122* ^Cre/+^ mice. The percentage of each receptor is shown in panel **(H)**, accordingly (n ≥6 for each group). All data represent at least three independent experiments. Statistical significance was determined by one-tailed Student’s t-test. *P < 0.05, **P < 0.01, and ***P < 0.001. Data are means ± SD.

We next detected NK cells in the *Tcf7* ^fl/fl^ *CD122* ^Cre/+^ mice. Similar to the *Tcf7* ^fl/fl^ *Vav* ^Cre/+^ mice, the frequency and numbers of total NK1.1^+^ cells were substantially reduced in BM, SP, and PBCs due to conditional targeting Tcf1 with *CD122*-Cre (**Figures 3A, B**). Accordingly, a blockade of the development from NKP to next stage was observed in BM from the *Tcf7* ^fl/fl^ *CD122* ^Cre/+^ mice (**Figures 3C, D**). In both BM and SP, the percentages of CD11b^low^ iNK cells and DP NK cells were severely reduced and the percentage of CD27^low^ mNK cells was elevated, whereas the absolute numbers of each subset were impaired corresponding to diminished total numbers of NK1.1^+^ cells in the *Tcf7* ^fl/fl^ *CD122* ^Cre/+^ mice (**Figures 3E, F**). The expression of Ly49 and KLRG1 receptors exhibited similar alteration in splenic NK cells from the *Tcf7* ^fl/fl^ *CD122* ^Cre/+^ mice in comparison with those in the *Tcf7* ^fl/fl^ *Vav* ^Cre/+^ mice, except the expression of Ly49G2 was not significantly changed (**Figures 3G, H**). Together, these data suggested that Tcf1 deficiency at NKP stage caused consistent phenotypic defects in NK cells lacking Tcf1 at HSC stage.

### *Tcf7* ^fl/fl^ *Ncr1* ^Cre/+^ Mice Show Modest Phenotypes and Distinct Expression Pattern of Ly49 Receptors in Splenic NK Cells

**Figure 4:**
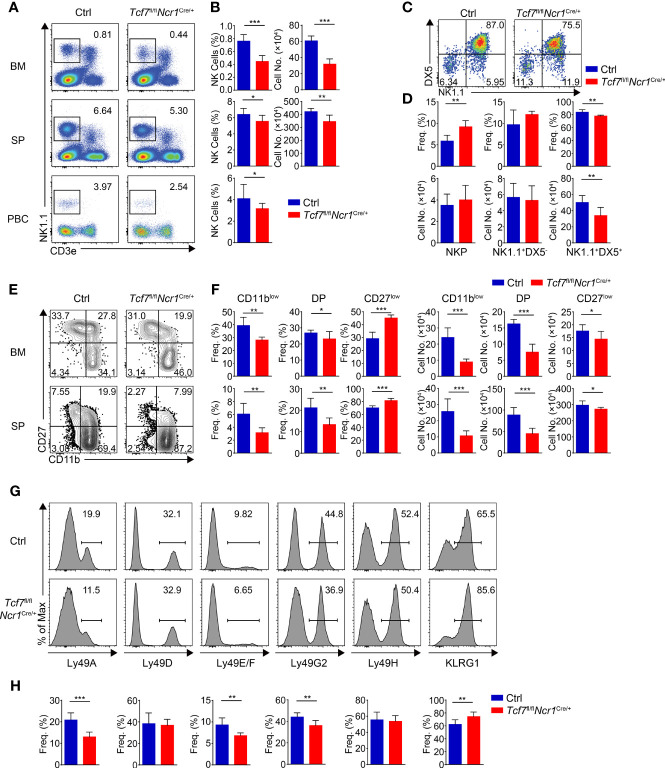
Lacking Tcf1 only in Ncr1^+^ cells leads to modest defects in NK cell development. **(A)** Flow cytometry analysis of BM, SP, and PBCs from Ctrl and *Tcf7* ^fl/fl^ *Ncr1* ^Cre/+^ mice. Representative pseudocolor plots showed total NK (CD3e^−^NK1.1^+^) cells from indicated tissue. **(B)** The frequency (left) and absolute numbers (right) of the NK cells from panel **(A)** are shown, accordingly (n ≥6 for each group). **(C)** Flow cytometry analysis of NKP cells in BM from Ctrl and *Tcf7* ^fl/fl^ *Ncr1* ^Cre/+^ mice. The CD3e^−^CD122^+^ cells were further analyzed by using NK1.1 and DX5 staining. Representative pseudocolor plots showed NKP (DX5^−^NK1.1^−^), DX5^−^NK1.1^+^, and DX5^+^NK1.1^+^ cells. **(D)** The percentages (upper) and absolute numbers (lower) of NKP, DX5^-^NK1.1^+^, and DX5^+^NK1.1^+^ cells from panel **(C)** are shown, accordingly (n ≥6 for each group). **(E)** Flow cytometry analysis of CD3e^−^NK1.1^+^ cell subsets from Ctrl and *Tcf7* ^fl/fl^ *Ncr1* ^Cre/+^ mice. The CD3e^−^NK1.1^+^ cells from BM or SP were further analyzed by using CD11b and CD27 staining. Representative contour plots showed CD11b^low^ (CD11b^−^CD27^+^), DP (CD11b^+^CD27^+^) and CD27^low^ (CD11b^+^ CD27^−^) NK cells. **(F)** The frequency (left) and absolute numbers (right) of CD11b^low^, DP, and CD27^low^ NK cells from panel **(E)** are shown, accordingly (n ≥6 for each group). **(G, H)** Representative histograms **(G)** showed the expression of Ly49A, Ly49D, Ly49E/F, Ly49G2, Ly49H, and KLRG1 in SP NK cells from Ctrl and *Tcf7* ^fl/fl^ *Ncr1* ^Cre/+^ mice. The percentage of each receptor is shown in panel **(H)**, accordingly (n ≥6 for each group). All data represent at least three independent experiments. Statistical significance was determined by one-tailed Student’s t-test. *P < 0.05, **P < 0.01, and ***P < 0.001. Data are means ± SD.

By analyzing *Tcf7* ^fl/fl^ *Ncr1* ^Cre/+^ mice, we found that the frequency and numbers of total NK1.1^+^ cells were significantly reduced in BM, SP, and PBCs in comparison to those from their littermate Ctrls (**Figures 4A, B**). The decreased frequency and numbers in BM NK cells from the *Tcf7* ^fl/fl^ *Ncr1* ^Cre/+^ mice were comparable with those in the *Tcf7* ^fl/fl^ *Vav* ^Cre/+^or *Tcf7* ^fl/fl^ *CD122* ^Cre/+^ mice, but the phenotypic defects on impaired frequency and numbers of NK cell in SP or PBCs were modest (**Figures 4A, B**). Similar to the *Tcf7* ^fl/fl^ *Vav* ^Cre/+^and *Tcf7* ^fl/fl^ *CD122* ^Cre/+^ mice, the frequency of NKP subset was notably increased and the percentage of CD3e^−^CD122^+^DX5^+^NK1.1^+^ cells was decreased in BM from the *Tcf7* ^fl/fl^ *Ncr1* ^Cre/+^ mice (**Figures 4C, D**). The absolute numbers of NKP cells were not altered, whereas the CD3e^−^CD122^+^DX5^+ ^NK1.1^+^ cells were diminished (**Figures 4C, D**). The similar defective phenotypes in frequency and numbers of CD11b^low^, DP, and CD27^low^ NK cells were exhibited in comparison with those from the *Tcf7* ^fl/fl^ *Vav* ^Cre/+^and *Tcf7* ^fl/fl^ *CD122* ^Cre/+^ mice (**Figures 4E, F**). For the expression of Ly49 receptors, we found that conditional ablation of Tcf1 in only Ncr1^+^ cells led to decreased expression of Ly49A, Ly49E/F, and Ly49G2 and increased expression of KLRG1, but no alteration in Ly49D. These results are in line with the findings from Dr. Barbara Kee’s group by using their *Tcf7* ^fl/fl^ *Ncr1* ^Cre/+^ strain (32), but not consistent with those from mice lacking Tcf1 at NKP stage, particularly in expression of Ly49D, Ly49E/F, and Ly49G2 (**Figures 4G, H**). Collectively, compared with the *Tcf7* ^fl/fl^ *Vav* ^Cre/+^ and *Tcf7* ^fl/fl^ *CD122* ^Cre/+^ mice, the *Tcf7* ^fl/fl^ *Ncr1* ^Cre/+^ mice exhibited similar phenotypes in BM NK cells but showed modest defects and distinct expression patterns of Ly49 receptors in splenic NK cells.

### Tcf1 Deficiency Results in Elevated Apoptosis in Distinct NK Subsets

**Figure 5:**
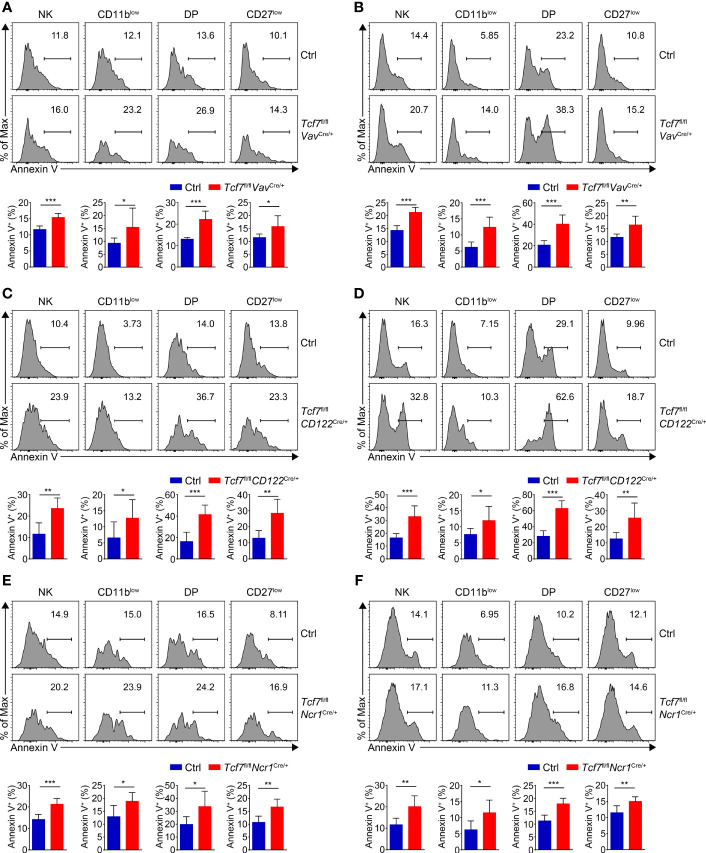
Analyzing the NK cell survival in the three mouse models. **(A)** Analyzing apoptosis of NK subsets in BM from Ctrl and *Tcf7* ^fl/fl^ *Vav* ^Cre/+^ mice. Representative histograms showed the Annexin V^+^ cells of CD11b^low^, DP, and CD27^low^ NK subsets (upper panel). Frequency of Annexin V^+^ cells in indicated subsets is shown (lower panel), accordingly (n ≥6 for each group). **(B)** Analyzing apoptosis of NK subsets in SP from Ctrl and *Tcf7* ^fl/fl^ *Vav* ^Cre/+^ mice. Representative histograms showed the Annexin V^+^ cells of CD11b^low^, DP, and CD27^low^ NK subsets (upper panel). Frequency of Annexin V^+^ cells in indicated subsets is shown (lower panel), accordingly (n ≥6 for each group). **(C)** Analyzing apoptosis of NK subsets in BM from Ctrl and *Tcf7* ^fl/fl^ *CD122* ^Cre/+^ mice. Representative histograms showed the Annexin V^+^ cells of CD11b^low^, DP, and CD27^low^ NK subsets (upper panel). Frequency of Annexin V^+^ cells in indicated subsets is shown (lower panel), accordingly (n ≥6 for each group). **(D)** Analyzing apoptosis of NK subsets in SP from Ctrl and *Tcf7* ^fl/fl^ *CD122* ^Cre/+^ mice. Representative histograms showed the Annexin V^+^ cells of CD11b^low^, DP, and CD27^low^ NK subsets (upper panel). Frequency of Annexin V^+^ cells in indicated subsets is shown (lower panel), accordingly (n ≥6 for each group). **(E)** Analyzing apoptosis of NK subsets in BM from Ctrl and *Tcf7* ^fl/fl^ *Ncr1* ^Cre/+^ mice. Representative histograms showed the Annexin V^+^ cells of CD11b^low^, DP, and CD27^low^ NK subsets (upper panel). Frequency of Annexin V^+^ cells in indicated subsets is shown (lower panel), accordingly (n ≥6 for each group). **(F)** Analyzing apoptosis of NK subsets in SP from Ctrl and *Tcf7* ^fl/fl^ *Ncr1* ^Cre/+^ mice. Representative histograms showed the Annexin V^+^ cells of CD11b^low^, DP, and CD27^low^ NK subsets (upper panel). Frequency of Annexin V^+^ cells in indicated subsets is shown (lower panel), accordingly (n ≥6 for each group). All data were pooled from at least two independent experiments. Statistical significance was determined by one-tailed Student’s t-test. *P < 0.05, **P < 0.01, and ***P < 0.001. Data are means ± SD.

Given impaired survival was found in NK cells from Tcf1 germline knockout mice due to excessive expression of GZMB (31), we next extended the apoptosis assay in distinct subsets of BM and SP NK cells from all three strains. We found the percentages of Annexin V^+^ cells were significantly increased in all NK subpopulations from the *Tcf7* ^fl/fl^ *Vav* ^Cre/+^, *Tcf7* ^fl/fl^ *CD122* ^Cre/+^, and *Tcf7* ^fl/fl^ *Ncr1* ^Cre/+^ mice (**Figures 5A**–**F**), indicating Tcf1 was crucial for survival of NK cells at each developmental phase.

### Tcf1 Controls the Transcription Programs for the Development of Early NK Cells

**Figure 6:**
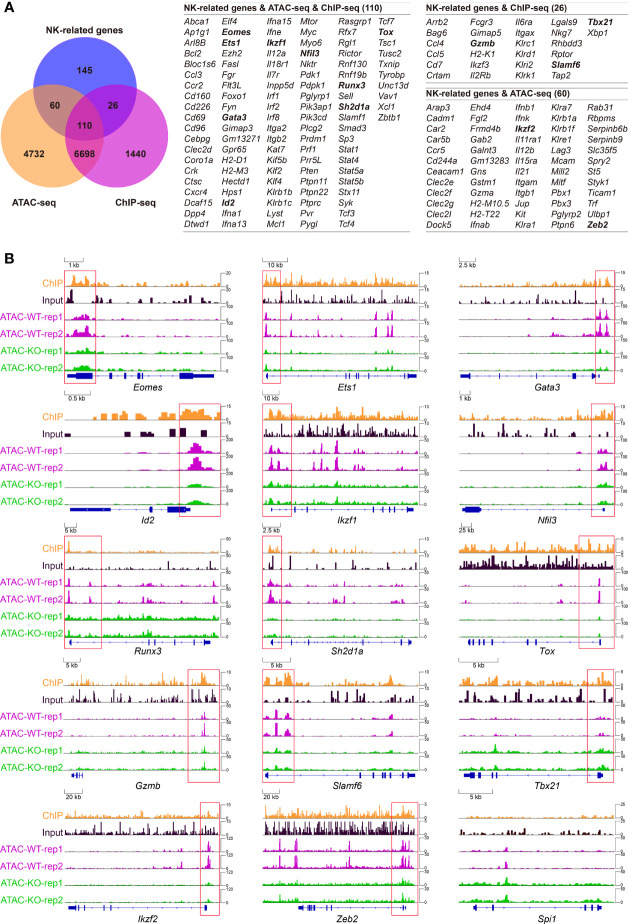
Combined analysis of high-throughput data showing Tcf1 associated NK regulatory program. **(A)** Left: Venn diagram showing the relationship among NK signature genes, Tcf1-dependent chromatin accessibility (differential ATAC-seq signals, GSE99159) and Tcf1 binding (differential enrich peaks from ChIP-seq analysis, GSE73239). The interested genes are shown in bold formatting. **(B)** The Integrated Genome Viewer (IGV) screenshots exhibiting ChIP-seq and ATAC-seq profiles in *Eomes*, *Ets1*, *Gata3*, *Id2*, *Ikzf1*, *Nfil3*, *RunX3*, *Sh2d1a*, *Slamf6*, *Tox*, *Tbx21*, *Ikzf2*, *Zeb2* and *Spi1* loci.

To further interrogate how Tcf1 modulates the key regulators in early NK cell development, we constructed a Tcf1-dependent transcriptional network by combining analysis of existing ATAC-seq and ChIP-seq data with NK regulatory genes. First, we established our “NK cell-related gene set” by combining the NK cell-associated genes collected from existing NK gene sets from three websites with established NK regulatory genes retrieved collected from published literatures (described in details in *Materials and Methods*). A Venn diagram analysis revealed that 110 of NK-cell related genes were directly bound by Tcf1 and their chromatin accessibility relied on Tcf1 (**Figure 6A**). In addition, 26 of NK-cell related genes which showed irrelevance with chromatin accessibility fell into the Tcf1 binding gene set, whereas 60 of NK cell-related genes exhibited differential chromatin accessibility in absence of Tcf1, but not directly bound by Tcf1 (**Figure 6A**). Based on previous knowledge, we further inspected a number of essential NK regulatory genes loci and analyzed their Tcf1 binding peaks and ATAC-seq peaks’ distribution, including *Eomes*, *Ets1*, *Gata3*, *Gzmb*, *Id2*, *Ikzf1*, *Ikzf2*, *Nfil3*, *RunX3*, *Sh2d1a*, *Slamf6*, *Tbx21*, *Tox*, *Zeb2*, and *Spi1*. At the promoter and regulatory regions of Tcf1 binding genes *Eomes*, *Ets1*, *Gata3*, *Id2*, *Ikzf1*, *Nfil3*, *RunX3*, *Sh2d1a*, and *Tox*, the alteration of chromatin accessibility was tightly associated with the absence of Tcf1 (**Figure 6B**). Meanwhile, *Gzmb*, *Slamf6*, and *Tbx21* were directly bound by Tcf1, whereas the chromatin accessibility did not show significant difference (**Figure 6B**). By contrast, we observed *Ikzf2* and *Zeb2* were indirectly regulated by Tcf1 due to the altered chromatin accessibility caused by Tcf1 deficiency (**Figure 6B**). Given that Spi1 has a critical function in early NK cell development, we exhibited its IGV for comparison even though neither was it bound by Tcf1 nor its chromatin accessibility was altered (**Figure 6B**). These results collectively suggested that Tcf1 is in charge of transcription programs corresponding to early NK cell development in a direct or indirect manner.

### Tcf1 Sustains the Expression of Multiple NK Cell Signature Genes in Distinct Developmental Phases

**Figure 7:**
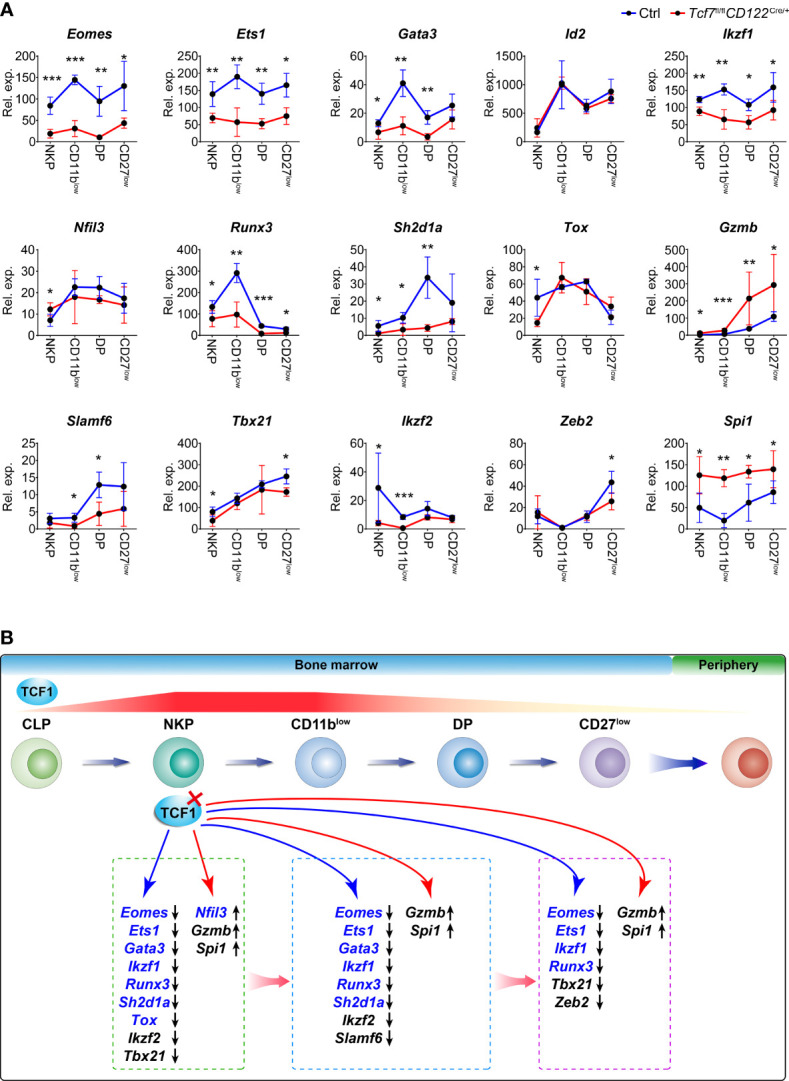
Tcf1 deficiency results in aberrant expression of multiple signature genes in distinct NK subsets. **(A)** RNA expression of the indicated genes was measured by qPCR in NKP, CD11b^low^, DP, and CD27^low^ subsets from Ctrl and *Tcf7* ^fl/fl^ *CD122* ^Cre/+^ mice. *Gapdh* was arbitrarily set to 1,000 and the relative expression in each subset was normalized, accordingly (n ≥3 for each group). All data represent at least two independent experiments. Statistical significance was determined by one-tailed Student’s t-test. *P < 0.05, **P < 0.01, and ***P < 0.001. Data are means ± SD. **(B)** Diagram working model showing the regulation of Tcf1 in NK cell development. HSC-derived progenitor initiates the continuous processes of NK lineage differentiation from CLP to CD27^low^ in BM before egress into periphery. The gene expression gradient of Tcf1 (encoded by *Tcf7*) throughout NK cell development is indicated by the striped line, and the red color density variation provides an approximation of the dynamic changes in *Tcf7* expression. The red cross mark represents the deletion of Tcf1 in NKP stage. The blue arrow line pointed to the downregulated genes in absence of Tcf1; the red arrow line pointed to the upregulated genes in absence of Tcf1. The Tcf1-binding genes from ChIP-seq data are highlighted in blue color. Differentially expressed genes due to Tcf1 deficiency are listed in the broken line circled areas which represented relative developmental phases. The black arrows following genes in the boxes indicate the downregulated expression or upregulated expression, respectively.

We next confirmed the expression of genes referred in **Figure 6B** in NKP, CD11b^low^, DP, and CD27^low^ cells from the *Tcf7* ^fl/fl^ *CD122* ^Cre/+^ mice by qPCR. The results indicated most of them were downregulated in all developmental stages, namely, *Eomes*, *Ets1*, *Gata3*, *Ikzf1*, *Ikzf2*, *Runx3*, *Sh2d1a*, and *Slamf6*, reflecting Tcf1 positively regulated their expression in NK cells (**Figure 7A**). Contrastingly, *Spi1* and *Gzmb* were upregulated at all developmental stages of NK cell in absence of Tcf1, implying Tcf1 negatively regulated their expression in NK cells (**Figure 7A**). *Nfil3* exhibited a slight elevation in NKP stage and its expression was slightly impaired in following developmental phases due to ablation of Tcf1 (**Figure 7A**). *Tox* was downregulated only in NKP cells caused by Tcf1 deficiency, but no significant alteration was observed in CD11b^low^, DP, and CD27^low^ cells (**Figure 7A**). The expression of *Tbx21* was significantly lower in Tcf1-deficient NKP and CD27^low^ cells than that from Ctrls, but only slightly downregulated in CD11b^low^ and DP subsets (**Figure 7A**). Despite the differential gene expression in NKP might be caused by the loss of NK progenitors in Tcf1-deficient mice, the expression of many genes exhibits the same trends in following more mature NK cells. It is worthy mentioning that the altered expression of Tox and Nfil3 was only detected in NKP cells, but no significant changes in the following stages, suggesting that we have to cautiously consider their aberrant expression in NKP stage due to Tcf1 deficiency. Based on our bioinformatic analysis above and the gene expression results generated from the *Tcf7* ^fl/fl^ *CD122* ^Cre/+^ mice, we constructed a working model to summarize the Tcf1 related regulatory programs linking to early NK cell development (**Figure 7B**). The dynamic expression of Tcf1 starts at CLP stage and reaches a peak in NKP and CD11b^low^ NK cells. During NK cell maturation, Tcf1 turns down its expression in DP subset and remains negligible level in CD27^low^ terminal mNK cells. All these four NK subpopulations exhibit the defective development when Tcf1 is deactivated prior to NKP phase. A number of essential NK governing genes are dysregulated in Tcf1-deficient NK cells, reflecting Tcf1 serves as their modulator in a direct or indirect manner. Thus, we identified the essential targets of Tcf1 and uncovered a Tcf1 associated regulatory network in maintaining early NK cell development.

## Discussion

Although Tcf1 is predominantly known for its critical functions in T cells, its biological functions extend to other immune cells, such as ILCs and NK cells (26, 42). For certain innate immune cell development, Tcf1 upregulates its expression in both early ILCs and NK progenitors and subsequently acts in promoting these cell commitment and differentiation (43). The importance of Tcf1 in NK cell was initially recognized for its role in acquisition of the Ly49A NK cell receptor (28). Then the Tcf1-dependent effects on Ly49 family receptors in both positive and negative ways were extensively explicated by Held and his colleagues (29, 30). An emerging study has indicated that Tcf1 guides NK cells through three stages of development, and protects mature NK cells from GZMB-dependent self-destruction (31). Recent work revealed that Tcf1 is an important target of the E protein Id2 transcriptional axis to program NK cell maturation (32). Despite increasing pieces of evidence that explored the crucial functions of Tcf1 in NK cell development and maturation, to date, the essential Tcf1-dependent target genes remain to be defined.

In this study, *Tcf7* ^fl/fl^ mice were crossed with *Vav*-Cre, *CD122*-Cre, or *Ncr1*-Cre mice to delete Tcf1 conditionally at HSC, NKP or iNK stage, respectively. These mouse models were applied to investigate the role of Tcf1 in NK cells excluding the effects from microenvironments at distinct developmental phases. We observed aberrant development of NK cells and substantially diminished NK cell numbers in BM from all three strains, which is consistent with previous findings in *Tcf7^−/−^* mice (31). The number of splenic CD3e*^−^* NK1.1^+^ cells were also notably impaired in the absence of Tcf1 before NKP stage. The splenic NK cells from the *Tcf7* ^fl/fl^ *Ncr1* ^Cre/+^ mice were also statistically reduced, but the phenotypic defects were much weaker than those in the other two strains. These results are in accordance with previous studies (31, 32). For different developmental subsets, we did not find a significant alteration in NKP numbers, but the cell numbers of all subsequent subpopulations were more or less diminished corresponding to the reduced total CD3e*^−^* NK1.1^+^ cells from all three strains. However, the *Tcf7* ^fl/fl^ *Ncr1* ^Cre/+^ mice exhibited more modest defects in NK cells from periphery. Given the NK cells from the *Tcf7^−/−^* mice showed normal proliferation and impaired survival due to overexpression of GZMB (31), we extended the apoptosis assay in CD11b^low^, DP, and CD11b^low^ cells. Our results reflected that all the NK subsets lacking Tcf1 exhibited increased apoptosis, indicating the Tcf1-dependent apoptosis in NK cells had no stage-specific feature.

To inspect whether conditional deletion of Tcf1 at distinct phases could result in different effects in acquisition of the Ly49 family NK cell receptors, we examined multiple Ly49 receptors in splenic NK cells from all three strains. Interestingly, we found that their expression was not quite consistent in the absence of Tcf1 at distinct developmental stages. Among them, Ly49A remained alike downregulation and Ly49H kept not changed from all three kinds of conditional knockout mice. Ly49D was downregulated in the *Tcf7* ^fl/fl^ *Vav* ^Cre/+^ and *Tcf7* ^fl/fl^ *CD122* ^Cre/+^ mice, but remained not changed in the *Tcf7* ^fl/fl^ *Ncr1* ^Cre/+^ mice. Ly49E/F was upregulated in the *Tcf7* ^fl/fl^ *Vav* ^Cre/+^ and *Tcf7* ^fl/fl^ *CD122* ^Cre/+^ mice, whereas it was downregulated in the *Tcf7* ^fl/fl^ *Ncr1* ^Cre/+^ mice. The expression of Ly49G2 was upregulated in the *Tcf7* ^fl/fl^ *Vav* ^Cre/+^ mice, not altered in the *Tcf7* ^fl/fl^ *CD122* ^Cre/+^ mice, and slightly downregulated in the *Tcf7* ^fl/fl^ *Ncr1* ^Cre/+^ mice. Our results in Ly49 receptors from the *Tcf7* ^fl/fl^ *Ncr1* ^Cre/+^ mice are in accordance with previous findings by using the same strategy to conditionally deactivate Tcf1 in NK cell with *Ncr1*-cre mice (32). However, the distinct expression patterns of Ly49D, Ly49E/F, and Ly49G2 were detected in the *Tcf7* ^fl/fl^ *Vav* ^Cre/+^ and *Tcf7* ^fl/fl^ *CD122* ^Cre/+^ mice, reflecting the stage-specific features of Tcf1 in regulating their expression. Therefore, further understanding of how Tcf1 dynamically controls acquisition of the Ly49 receptors is required in future studies.

Although previous studies established the importance of Tcf1 in NK cell development and described the severe phenotypes in NK cells by using *Tcf7^−/−^* and *Tcf7* ^fl/fl^ *Ncr1* ^Cre/+^ mice, the essential targets downstream Tcf1 have not been identified. Owing to a large number of transcription factors controlling early developmental programs of NK cells parallel to those of CD8^+^ T cells, we performed further bioinformatic analysis by combining the NK cell-related gene set with published high quality Tcf1 ChIP-seq in CD8^+^ T cells (44) and ATAC-seq in DP thymocytes (45), which reflected the Tcf1-bound genes and the altered chromosome accessibility in the absence of Tcf1, respectively. As a result, a large number of essential NK regulators fell into the Tcf1 binding gene set and/or exhibit alteration of their chromatin accessibility, suggesting Tcf1 directly or indirectly affects their expression. Following the clue from bioinformatic analysis, we then validated their mRNA abundances in NKP, CD11b^low^, DP, and CD11b^low^ cells from the *Tcf7* ^fl/fl^ *CD122* ^Cre/+^ mice. Similar to a previous report by Jeevan-Raj and colleagues, the expression of *Gzmb* was upregulated and *Id2* was not altered, whereas *Nfil3* and *Tbx21* expression exhibited minor changes (31). Strikingly, we found that a series of positive NK regulators were downregulated in the absence of Tcf1 along with step-wise developmental stages, namely, *Eomes*, *Ets1*, *Gata3*, *Ikzf1*, *Ikzf2*, *Runx3*, *Sh2d1a*, and *Slamf6*. In addition, the *Spi1* expression was remarkably elevated in all subsets though it was not a direct target of Tcf1. The aberrant expression of these NK regulatory genes provided new evidences linking to the severe defects in NK cells lacking Tcf1. Overall, our data identified the essential targets of Tcf1 and uncovered a Tcf1-dependent regulatory transcription network in regulating NK cell development.

## Data Availability Statement

The original contributions presented in the study are included in the article/**Supplementary Material**. Further inquiries can be directed to the corresponding authors.

## Ethics Statement

The animal study was reviewed and approved by the China Agricultural University Laboratory Animal Welfare and Animal Experiment Ethics Review Committee.

## Author Contributions

ShuyY designed and supervised the experiments with constructive suggestions. JuL, ZW, and SH performed the major experiments. JL and ZW analyzed the overall data and made figures. ZW and YajZ analyzed the high throughput data. FW, YY, YajZ, YanZ, WG, GY, XM, JiL, FC, and YK assisted the overall experiments. ShuyY, YK, ShunY, and SH wrote the manuscript with the revision from all authors. All authors contributed to the article and approved the submitted version.

## Funding

This work was supported in part by grants from the National Key Research and Development Program of China (2017YFA0104401 and 2016YFC1101102), the National Natural Scientific Foundation of China (32130039, 31970831, 81770207, 31630038, and 82170230), the Chinese Universities Scientific Fund (2021TC087), and the Project for Extramural Scientists of State Key Laboratory of Agrobiotechnology from the China Agricultural University (2021SKLAB6-3 and 2021SKLAB6-4).

## Conflict of Interest

The authors declare that the research was conducted in the absence of any commercial or financial relationships that could be construed as a potential conflict of interest.

## Publisher’s Note

All claims expressed in this article are solely those of the authors and do not necessarily represent those of their affiliated organizations, or those of the publisher, the editors and the reviewers. Any product that may be evaluated in this article, or claim that may be made by its manufacturer, is not guaranteed or endorsed by the publisher.
