# Supplementary material for: Tcf1 Sustains the Expression of Multiple Regulators in Promoting Early Natural Killer Cell Development
Source: Front Immunol. 2021 Nov 30;12:791220. doi: 10.3389/fimmu.2021.791220 (PMC8669559; doi:10.3389/fimmu.2021.791220)
Supplement: Supplementary file 1 [file Table_1.docx]

**Supplementary Table 1: Primers for qPCR and genotyping**

| **Name** | **Sequence (5’→3’)** |
| --- | --- |
| **Primers for qPCR** |  |
| RT-*Eomes*-F | TCCTAACACTGGCTCCCACT |
| RT-*Eomes-*R | GTCACTTCCACGATGTGCAG |
| RT-*Ets1*-F | TCCTATCAGCTCGGAAGAACTC |
| RT-*Ets1*-R | TCTTGCTTGATGGCAAAGTAGTC |
| RT-*Gapdh*-F | ATGGTGAAGGTCGGTGTGAA |
| RT- *Gapdh* -R | GTCGTTGATGGCAACAATCTCC |
| RT-*Gata3*-F | CTTATCAAGCCCAAGCGAAG |
| RT-*Gata3*-R | CATTAGCGTTCCTCCTCCAG |
| RT-*Gzmb*-F | CAAAGACCAAACGTGCTTCC |
| RT-*Gzmb*-R | CTCAGCTCTAGGGACGATGG |
| RT-*Id2*-F | GTCCTTGCAGGCATCTGAAT |
| RT-*Id2*-R | TTCAACGTGTTCTCCTGGTG |
| RT-*Ikzf1*-F | CACAACGAGATGGCAGAAGA |
| RT-*Ikzf1*-R | CTGACAGGCACTTGTCTCCA |
| RT-*Ikzf2*-F | GAGCCGTGAGGATGAGATCAG |
| RT-*Ikzf2*-R | CTCCCTCGCCTTGAAGGTC |
| RT-*Nfli3*-F | CAGTGCAGGTGACGAACATT |
| RT-*Nfli3*-R | TTCCACCACACCTGTTTTGA |
| RT-*Runx3*-F | AGGGAAGAGTTTCACGCTCA |
| RT-*Runx3*-R | AGGCCTTGGTCTGGTCTTCT |
| RT- *Sh2d1a*-F | CTGGATGGAAGCTATCTGCT |
| RT- *Sh2d1a*-R | GAATCTCTTCTCCCTGTGGG |
| RT-*Slamf6*-F | CCCTGGAATGCAGTATGGTT |
| RT-*Slamf6*-R | GCTCTGGGAGGACTCTGGAT |
| RT-*Spi1*-F | GGGCATCCAGAAGGGCAA |
| RT-*Spi1*-R | GGTAGGTGAGCTTCTTCTTGA |
| RT-*Tbx21*-F | CAACAACCCCTTTGCCAAAG |
| RT-*Tbx21*-R | TCCCCCAAGCAGTTGACAGT |
| RT-*Tox*-F | GAGGATGCCTCCAAGATCAA |
| RT-*Tox*-R | GCCTGGGTATCACGAAAGAA |
| RT-*Zeb2*-F | CCAGAGGAAACAAGGATTTCAG |
| RT-Zeb2-R | AGGCCTGACATGTAGTCTTGTG |
| **Primers for genotyping** |  |
| *CD122*-F | CAGAGCAGCTTTGACAACCCAAACG |
| *CD122*-R | TGCTTCACAGAAAAACCCACCCCAG |
| *CD122*-cre | CATACAATGGGGTACCTTCTGGGC |
| *Ncr1*-F | AATTGGTCTGGCATGCATAATC |
| *Ncr1*-R | AGTAGGAAGGAAATATTCCCATGG |
| *Ncr1*-cre | CACACCGGCCTTATTCCAAG |
| *Tcf7*-F | AGCTGAGCCCCTGTTGTAGA |
| *Tcf7*-R1 | TTCTTTGACCCCTGACTTGG |
| *Tcf7*-R2 | CAACGAGCTGGGTAGAGGAG |
| *Vav*-F | AGATGCCAGGACATCAGGAACCTG |
| *Vav*-R | ATCAGCCACACCAGACACAGAGATC |
